# Associations between per- and polyfluoroalkyl substance exposure and the prevalence of myopia in adolescents: the mediating role of serum albumin

**DOI:** 10.1265/ehpm.25-00023

**Published:** 2025-06-27

**Authors:** Xuewei Li, Xiaodong Chen, Yixuan Zhang, Tonglei Zheng, Lvzhen Huang, Yan Li, Kai Wang

**Affiliations:** 1 Department of Ophthalmology, Peking University People’s Hospital, Beijing, China; 2 Institute of Medical Technology, Peking University Health Science Center, Beijing, China; 3 Beijing Key Laboratory of Ocular Disease and Optometry Science, Peking University People’s Hospital, Beijing, China; 4 College of Optometry, Peking University Health Science Center, Beijing, China

**Keywords:** Per- and polyfluoroalkyl substances, National Health and Nutrition Examination Survey, Myopia, Albumin

## Abstract

**Background:**

The objective of this study was to investigate the potential link between myopia in adolescents and exposure to per- and polyfluoroalkyl substances (PFASs).

**Methods:**

This investigation included 1971 subjects with accessible PFAS level data, myopia status, and associated variables from four cycles of the National Health and Nutritional Examination Survey (NHANES). The investigation focused on specific PFAS compounds found in the serum, including perfluorohexane sulfonate (PFHxS), perfluorononanoic acid (PFNA), perfluorooctanoic acid (PFOA), and perfluorooctane sulfonic acid (PFOS), chosen for their frequent detection. Owing to the skewed nature of the PFAS level data, the PFAS levels were log-transformed (Ln-PFAS) prior to analysis. Logistic regression, restricted cubic spline modeling, subgroup analysis, and sensitivity analysis were used to examine the associations between exposure to PFASs and the onset of myopia.

**Results:**

PFOA levels were significantly associated with myopia risk (OR: 1.33; 95% CI: 1.05–1.69; P = 0.019). More specifically, with respect to the first quartile, the second quartile (OR_Q2_: 1.69; 95% CI: 1.16–2.46; P = 0.007), third quartile (OR_Q3_: 1.45; 95% CI: 1.03–2.03; P = 0.035), and highest quartile (OR_Q4_: 1.58; 95% CI: 1.12–2.21; P = 0.010) of participants presented with increased myopia risk. Mediation analysis revealed that PFOA and myopia risk were partially mediated by serum albumin (ALB), with a mediation percentage of 22.48% (P = 0.008). A nonlinear inverted U-shaped relationship was identified between the level of PFOA and myopia risk (P for nonlinearity = 0.005).

**Conclusion:**

Our findings suggest a potential link between exposure to PFOA and the likelihood of myopia development in young individuals and a mediating effect of serum ALB on this relationship. Notably, PFOA was identified as a key PFAS significantly contributing to the observed link between PFAS exposure and myopia risk. The potential threat of PFOA to myopia should be examined further.

**Supplementary information:**

The online version contains supplementary material available at https://doi.org/10.1265/ehpm.25-00023.

## Graphical Abstract

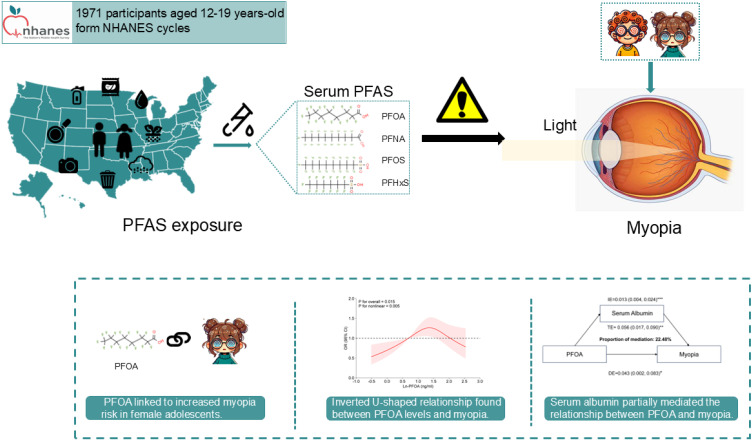

## 1. Introduction

Myopia, commonly referred to as short- or near-sightedness, is a prevalent visual disorder that usually emerges in childhood or early adulthood [1, 2]. In recent years, the global prevalence of myopia has increased significantly, with a particularly sharp increase observed among children and adolescents in East Asian countries [3]. This escalation is of great concern, as by the year 2050, approximately 49.8% of the global population, equivalent to approximately 4.76 billion individuals, are expected to be afflicted by myopia [4]. Alongside the increasing incidence of myopia, the age of onset has notably shifted lower, especially in East Asia [5, 6]. In addition to general inconveniences in daily life, myopia can seriously increase the risk of eye diseases, such as myopic macular degeneration, retinal detachment, glaucoma and cataracts, all of which can lead to permanent loss of vision, seriously endangering the individual’s life [7, 8]. Therefore, it is necessary to control the progression of myopia during adolescence.

Myopia is caused by abnormal elongation of the vitreous cavity involving the regulation of the extracellular matrix (ECM) and scleral remodeling by genetic [9], environmental [10], and behavioral factors [11]. These changes lead to reduced collagen accumulation and altered ECM composition, triggering myopia [12–14]. In contrast with nonmyopic eyes, myopic eyes contain relatively high levels of standard inflammatory cytokines in the aqueous (vitreous) humor [15–17]. In addition, experiments have demonstrated that myopic eyes show relatively high activation of inflammatory signaling pathways [18, 19]. The specific degree of ocular inflammation and oxidative stress imbalance may contribute to the development of myopia [20]. Ocular inflammatory conditions, such as allergic conjunctivitis [21], uveitis [22], and scleritis [23], may contribute to the progression of myopia by increasing the degree of ocular inflammation. Although the specific causes of myopia remain unclear, a growing number of studies have suggested that environmental factors are involved [24–26]. Several studies have highlighted the impact of environmental pollutants on eye health; for example, PM2.5 [27], ozone [28], and nitrogen oxides [29] all contribute to the development of myopia, which maybe affected by inflammation and oxidative stress.

Per- and polyfluoroalkyl substances (PFASs), known as “forever chemicals,” are widely used synthetic compounds valued for their stability and resistance to degradation [30, 31]. However, their persistence in the environment leads to widespread human exposure through contaminated water, food, and air [32–34]. PFASs have been linked to endocrine disruption [35, 36], carcinogenicity [37, 38], and neurotoxicity [39], with oxidative stress and inflammation proposed as key mechanisms [40]. While their health risks are well-documented, few studies have examined their potential role in myopia development. Previous studies have demonstrated that PFOA exposure increases ROS and inflammatory markers such as IL-6 [41–44]. Similarly, elevated levels of oxidative stress and inflammation markers, including IL-6 and MMP-2, have been observed in myopic eyes [45–47]. Given the increasing prevalence of myopia, our study aims to bridge this research gap by investigating the relationship between serum PFAS levels and myopia risk.

In this study, we conducted an observational research-based survey with nationally representative information from the National Health and Nutritional Examination Survey (NHANES) to explore the possible associations between serum PFAS concentrations and myopia progression in adolescents. Additionally, we investigated whether the serum ALB concentration might serve as a potential mediator of the relationship between PFAS exposure and myopia. The objective of this study was to gain deeper epidemiological insights into the toxicological effects of PFASs and their possible impact on myopia, which could guide the development of strategies for disease prevention and management.

## 2. Methods

### 2.1 Data collection and study population

**Fig. 1:**
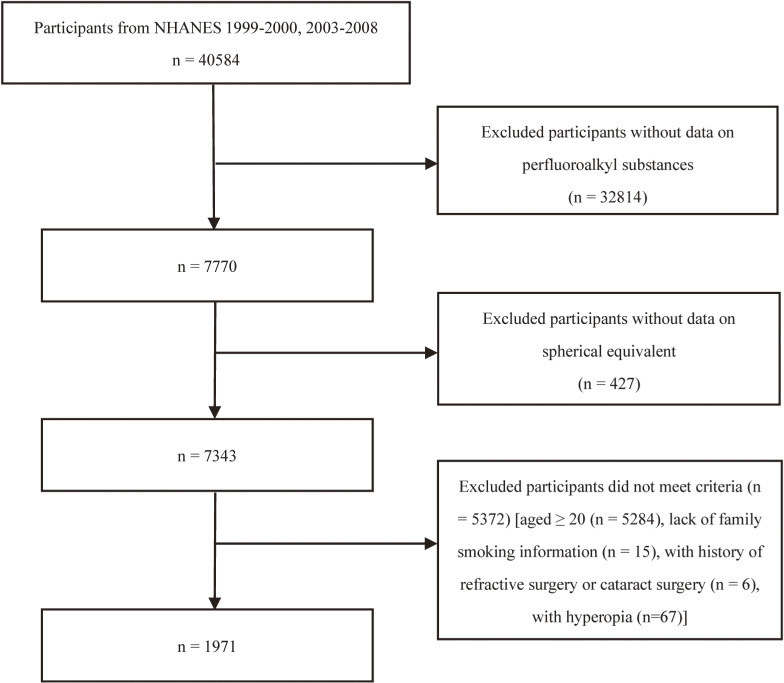
Flow diagram illustrating exclusion criteria and final number of individuals included at each stage.

This study involved data from the NHANES from 1999–2008. Operated by the National Center for Health Statistics (NCHS), part of the Centers for Disease Control (CDC), the NHANES includes in-depth interviews paired with physical examinations and is designed to represent the noninstitutionalized civilian U.S. population [33]. For this study, (Fig. 1), 40,584 individuals across four cycles of the NHANES (1999–2000, 2003–2004, 2005–2006, and 2007–2008 cycles) were included. Participants who lacked serum PFAS concentration data (n = 32,814) or spherical equivalent refraction (SE) measurements (n = 427), who were 20 years of age or older (n = 5,284), who lack smoking information (n = 15), who had history of refractive surgery or cataract surgery (n = 6), or had hyperopia (n = 67) were excluded. After the exclusion criteria were applied, the final cohort consisted of 1,971 participants. In accordance with the ethical guidelines of the Declaration of Helsinki, all volunteers provided informed consent in writing. The research methodologies were thoroughly reviewed and subsequently confirmed by the NCHS Institutional Review Board.

### 2.2 Myopia assessments

Refractive error was measured with a Nidek Auto Refractor Model ARK-760 (Nidek Co., Ltd., Gamagori, Japan), which recorded the median values of three key objective parameters for participants: sphere, cylinder, and axis. The evaluation was conducted on both eyes after any corrective lenses had been removed. The spherical equivalent refraction (SER) was determined by adding the sphere value to one-half of the cylinder value under noncycloplegic conditions. Myopia was defined as an SER less than −1.0 diopters (D), while mild myopia, moderate myopia, and high myopia were defined as −3D < SER ≤ −1D, −5D < SER ≤ −3D, or SER ≤ −5D, respectively, according to the guidelines in previous studies [48, 49].

### 2.3 PFAS assessments

Serum PFAS concentrations in participants older than 12 were determined through an online solid-phase extraction process, followed by high-performance liquid chromatography–turbo ion spray tandem mass spectrometry. This approach was employed on a randomly chosen subset comprising approximately one-third of the entire sample. Owing to the low detection rates of some PFAS concentrations, four commonly detected PFAS compounds, including perfluorooctanoic acid (PFOA), perfluorohexane sulfonate (PFHxS), perfluorononanoic acid (PFNA), and perfluorooctane sulfonic acid (PFOS), were examined in this study. When PFAS levels were undetectable, they were given a value calculated as the limit of detection (LoD) divided by the square root of two. Table S1 lists the LoD values of the four PFAS compounds in the NHANES cycles.

### 2.4 Covariates

Several covariates were incorporated to mitigate the influence of potential confounding factors: age, sex, race (Mexican American, other Hispanic, non-Hispanic White, non-Hispanic Black, or other), family income–poverty ratio (FIR), body mass index (BMI, kg/m^2^). Considering previous research on the impact of education [50] and smoking [51] on myopia, education level (less than 9th grade, high school, or high school graduate and above), and smoking status of family members were also included.

### 2.5 Statistical analyses

All analyses in this study adhered to the CDC guidelines for complex oversampling data. The analyses were based on weighted estimates with sample weights [52].

Descriptive statistics for demographic data are expressed in terms of mean values with their corresponding standard errors (SE) or as proportions. We analyzed group differences in baseline characteristics with either the t test or the design-adjusted Rao–Scott Pearson χ_2_ test. To address missing data in the covariates, multiple imputation was applied for the BMI and FIR (n = 127) [53]. We generated five imputation datasets and selected one for further analysis. Considering the skewed nature of the PFAS concentration data, we included the natural log-transformed PFAS values (Ln-PFAS) as continuous covariates within the regression models. We employed two weighted logistic regression models to calculate the odds ratios and their respective 95% confidence intervals. PFAS serum concentrations were also analyzed in quartiles. The basic model was free from any adjustments, whereas Model 1 incorporated adjustments for all covariates. Subgroup analyses were conducted, stratified by sex, race, and education level. Restricted cubic spline (RCS) analysis was conducted to investigate the nonlinear correlation between PFAS exposure and the risk of developing myopia.

The RCS model incorporated the natural Ln-PFAS as a continuous predictor and was adjusted according to the covariates employed in Model 1. The RCS analysis included knots positioned at the 10th, 50th, and 90th percentiles of the distribution for Ln-PFAS levels. Mediation analysis was conducted via the quasi-Bayesian Monte Carlo method with the R package “mediation”. We hypothesized that the serum ALB concentration mediates the relationship between PFAS exposure and the risk of myopia. The direct effect (DE) was the impact of PFAS exposure on myopia without affecting the serum ALB concentration. The indirect effect (IE) was the impact of PFAS exposure on myopia through the serum ALB concentration. The total effect (TE) was the sum of IE and DE. The formula for calculating the proportion of indirect effects was (IE divided by TE) × 100%. Considering the effects of contact lenses (CLs) on PFAS levels, we conducted a sensitivity analysis after excluding those participants who wore CLs (n = 142). R software 4.3.2 was used for statistical analysis, and a two-tailed P value less than 0.05 indicated statistical significance.

## 3. Results

### 3.1 Study population

A total of 1,971 participants aged 12–19 year across four NHANES cycles (1999–2000, 2003–2004, 2005–2006, 2007–2008) were ultimately included in this study, 582 individuals (29.5%) had myopia. of which 380 had mild myopia, 137 had moderate myopia, and 65 had high myopia. There were no notable differences in age, sex, race, FIR, BMI, and education level between participants with myopia and those with normal vision (P > 0.05). However, the percentage of family members who smoked was lower in the myopia group than in the normal vision group (P = 0.004). Compared with those with normal vision, participants with myopia had slightly higher PFOA levels (P = 0.064). Table 1 provides a detailed summary of the demographic and lifestyle characteristics of the participants in this study.

**Table 1:** Characteristics of all study participants

	**All** **(n = 1,971)**	**Normal vision** **(n = 1,389)**	**Myopia** **(n = 582)**	**P value**
Age, years	15.5 ± 2.2	15.5 ± 2.2	15.6 ± 2.3	0.451
Sex				0.761
Male	1029 (51.6)	745 (51.9)	284 (50.9)	
Female	942 (48.4)	644 (48.1)	298 (49.1)	
Race				0.412
Non-Hispanic White	508 (62.6)	356 (63.5)	152 (60.6)	
Non-Hispanic Black	602 (14.2)	433 (14.1)	169 (14.2)	
Other	861 (23.2)	600 (22.4)	261 (25.2)	
Family income-poverty ratio	2.5 ± 1.6	2.5 ± 1.6	2.5 ± 1.6	0.991
BMI, kg/m^2^	23.8 ± 5.9	23.6 ± 5.7	24.3 ± 6.4	0.080
Education level				0.202
Less than 9th Grade	1129 (56.6)	815 (58.0)	314 (53.6)	
High school and above	842 (43.4)	574 (42.0)	268 (46.4)	
Family member smoking				0.004
Yes	420 (21.0)	317 (23.5)	103 (15.3)	
No	1551 (79.0)	1072 (76.5)	479 (84.7)	
Serum albumin, g/dl	4.4 (0.3)	4.4 ± 0.3	4.5 ± 0.3	0.018
SER	−0.9 ± 1.7	−0.0 ± 0.4	−2.9 ± 1.9	<0.001
PFHxS, Median (IQR), ng/ml	2.3 (3.4)	2.4 (3.2)	2.2 (3.7)	0.933
PFNA, Median (IQR), ng/ml	0.9 (0.7)	0.9 (0.7)	0.9 (0.8)	0.444
PFOA, Median (IQR), ng/ml	4.0 (2.4)	4.0 (2.5)	4.1 (2.2)	0.064
PFOS, Median (IQR), ng/ml	15.7 (13.6)	15.5 (13.6)	16.6 (13.3)	0.835

Abbreviations: BMI, body mass index; SE, spherical equivalent; PFHxS, perfluorohexane sulfonate; PFNA, perfluorononanoic acid; PFOA, perfluorooctanoic acid; PFOS, perfluorooctane sulfonic acid

### 3.2 Association between PFAS exposure and myopia

After accounting for the abovementioned factors, the concentration of PFOA as a continuous variable was significantly correlated with the likelihood of myopia (OR: 1.33; 95% CI: 1.05–1.69; P = 0.019). In the analysis of PFOA levels across quartiles, a significant relationship between the second (Q2), third (Q3), and fourth (Q4) quartiles and myopia was identified with respect to the first quartile (OR_Q2_: 1.69; 95% CI: 1.16–2.46; P = 0.007, OR_Q3_: 1.45; 95% CI: 1.03–2.03; P = 0.035, OR_Q4_: 1.58; 95% CI: 1.12–2.21; P = 0.010) (Table 2).

**Table 2:** Logistic regression analysis of the association between PFAS exposure and myopia.

	**Crude (OR, 95% CI)**	**P value**	**Model 1 (OR, 95% CI)**	**P value**
PFHxS level				
Ln-PFHxS level	0.98 (0.86, 1.10)	0.605	0.99 (0.87, 1.13)	0.907
Q1 (<1.2)				
Q2 (1.2–2.4)	1.22 (0.85, 1.76)	0.277	1.27 (0.90, 1.79)	0.186
Q3 (2.4–4.7)	0.80 (0.53, 1.22)	0.298	0.86 (0.56, 1.32)	0.488
Q4 (≥4.7)	1.12 (0.79, 1.59)	0.529	1.18 (0.81, 1.71)	0.389
PFNA level				
Ln-PFNA level	1.11 (0.94, 1.33)	0.221	1.13 (0.93, 1.37)	0.210
Q1 (<0.6)				
Q2 (0.6–0.9)	1.13 (0.75, 1.71)	0.548	1.16 (0.75, 1.77)	0.495
Q3 (0.9–1.312)	0.87 (0.57, 1.35)	0.538	0.91 (0.59, 1.41)	0.664
Q4 (≥1.312)	1.23 (0.82, 1.84)	0.309	1.26 (0.82, 1.95)	0.291
PFOA level				
Ln-PFOA level	**1.26 (1.03, 1.55)**	**0.028**	**1.33 (1.05, 1.69)**	**0.019**
Q1 (<3.0)				
Q2 (3.0–4.0)	**1.66 (1.12, 2.45)**	**0.012**	**1.69 (1.16, 2.46)**	**0.007**
Q3 (4.0–5.4)	1.37 (0.97, 1.91)	0.069	**1.45 (1.03, 2.03)**	**0.035**
Q4 (≥5.4)	**1.46 (1.08, 1.98)**	**0.015**	**1.58 (1.12, 2.21)**	**0.010**
PFOS level				
Ln-PFOS level	0.99 (0.80, 1.23)	0.954	1.08 (0.85, 1.37)	0.510
Q1 (<10.4)				
Q2 (10.4–16.0)	0.89 (0.56, 1.40)	0.609	0.97 (0.61, 1.53)	0.881
Q3 (16.0–24.4)	1.13 (0.75, 1.69)	0.561	1.26 (0.83, 1.91)	0.265
Q4 (≥24.4)	0.99 (0.66, 1.50)	0.975	1.16 (0.74, 1.82)	0.511

Abbreviations: PFHxS, perfluorohexane sulfonate; PFNA, perfluorononanoic acid; PFOA, perfluorooctanoic acid; PFOS, perfluorooctane sulfonic acid.

The crude model was not adjusted for any variables. Model 1 was adjusted for age, sex, ethnicity, educational attainment, the family’s income–poverty ratio, BMI, and the smoking habits of family members.

After stratified by myopia severity, PFNA (OR: 1.99; 95% CI: 1.19–3.33; P = 0.010) and PFOA (OR: 1.61; 95% CI: 1.08–2.38; P = 0.019) were significantly correlated with high myopia risk (Table 3).

**Table 3:** Logistic regression analysis of the association between PFAS exposure and myopia severity.

	**OR (95%CI)**	**P-value**
Mild myopia		
PFHxS	1.05 (0.89, 1.24)	0.575
PFNA	1.05 (0.81, 1.35)	0.712
PFOA	1.26 (0.92, 1.73)	0.138
PFOS	1.17 (0.86, 1.59)	0.303
Moderate myopia		
PFHxS	0.95 (0.72, 1.26)	0.714
PFNA	1.04 (0.67, 1.60)	0.865
PFOA	1.38 (0.94, 2.02)	0.095
PFOS	1.07 (0.72, 1.59)	0.715
High myopia		
PFHxS	0.84 (0.67, 1.06)	0.145
PFNA	**1.99 (1.19, 3.33)**	**0.010**
PFOA	**1.61 (1.08, 2.38)**	**0.019**
PFOS	0.77 (0.42, 1.40)	0.388

Note: PFAS, perfluoroalkyl substances; PFHxS, perfluorohexane sulfonate; PFNA, per fluorononanoic acid; PFOA, perfluorooctanoic acid; PFOS, perfluorooctane sulfonic acid

Model was adjusted for age, sex, race, education level, family income-poverty ratio, BMI, and smoking status of family members.

### 3.3 Subgroup analyses

The results of the subgroup analyses (stratified by sex, ethnicity, and educational attainment) are shown in Table 4. An important relationship was observed between the risk of myopia and PFOA exposure in women in the sex-stratified analysis (OR: 1.71; 95% CI: 1.19–2.46; P = 0.005). Similarly, in the ethnicity-stratified analysis, PFOA exposure was significantly related to the risk of myopia in non-Hispanic Whites (OR: 1.88; 95% CI: 1.31–2.70; P = 0.001). Finally, in the education-stratified analysis, PFOA exposure was significantly related to the risk of myopia among individuals with lower educational levels (OR: 1.46; 95% CI: 1.02–2.08; P = 0.040).

**Table 4:** Subgroup analyses

	**ln-PFHxS**		**ln-PFNA**		**ln-PFOA**		**ln-PFOS**	
			
	**OR (95% CI)**	**P value**	**OR (95% CI)**	**P value**	**OR (95% CI)**	**P value**	**OR (95% CI)**	**P value**
Age								
<16	1.00 (0.83, 1.21)	0.981	1.33 (0.98, 1.82)	0.070	1.29 (0.87, 1.91)	0.196	1.02 (0.70, 1.48)	0.926
≥16	0.99 (0.82, 1.19)	0.901	0.95 (0.71, 1.26)	0.695	1.35 (0.96, 1.91)	0.084	1.11 (0.85, 1.45)	0.425
Sex								
Male	0.96 (0.79, 1.16)	0.639	1.01 (0.78, 1.33)	0.913	1.04 (0.66, 1.63)	0.860	1.08 (0.74, 1.57)	0.697
Female	1.04 (0.85, 1.26)	0.711	1.27 (0.95, 1.69)	0.102	**1.71 (1.19, 2.46)**	**0.005**	1.08 (0.75, 1.56)	0.668
Race								
Non-Hispanic White	1.05 (0.87, 1.27)	0.572	1.30 (0.96, 1.76)	0.084	**1.88 (1.31, 2.70)**	**0.001**	1.29 (0.85, 1.95)	0.216
Non-Hispanic Black	0.98 (0.78, 1.23)	0.834	0.87 (0.57, 1.32)	0.490	0.92 (0.69, 1.24)	0.582	0.82 (0.61, 1.09)	0.160
Other Race	0.86 (0.68, 1.09)	0.204	1.00 (0.63, 1.37)	0.986	0.91 (0.61, 1.36)	0.643	0.89 (0.65, 1.22)	0.451
Education								
Less than 9th Grade	1.02 (0.86, 1.21)	0.838	1.21 (0.91, 1.61)	0.177	**1.46 (1.02, 2.08)**	**0.040**	1.04 (0.74, 1.47)	0.823
High school and above	0.96 (0.80, 1.16)	0.686	1.03 (0.79, 1.35)	0.797	1.16 (0.81, 1.66)	0.403	1.08 (0.81, 1.45)	0.597

Abbreviations: PFHxS, perfluorohexane sulfonate; PFNA, perfluorononanoic acid; PFOA, perfluorooctanoic acid; PFOS, perfluorooctane sulfonic acid.

Note: Adjustments were made for factors including age, sex, ethnicity, educational attainment, FIR, BMI, and the smoking habits of family members but not the variable used for grouping

### 3.4 Dose-response relationships between PFAS exposure and myopia

**Fig. 2:**
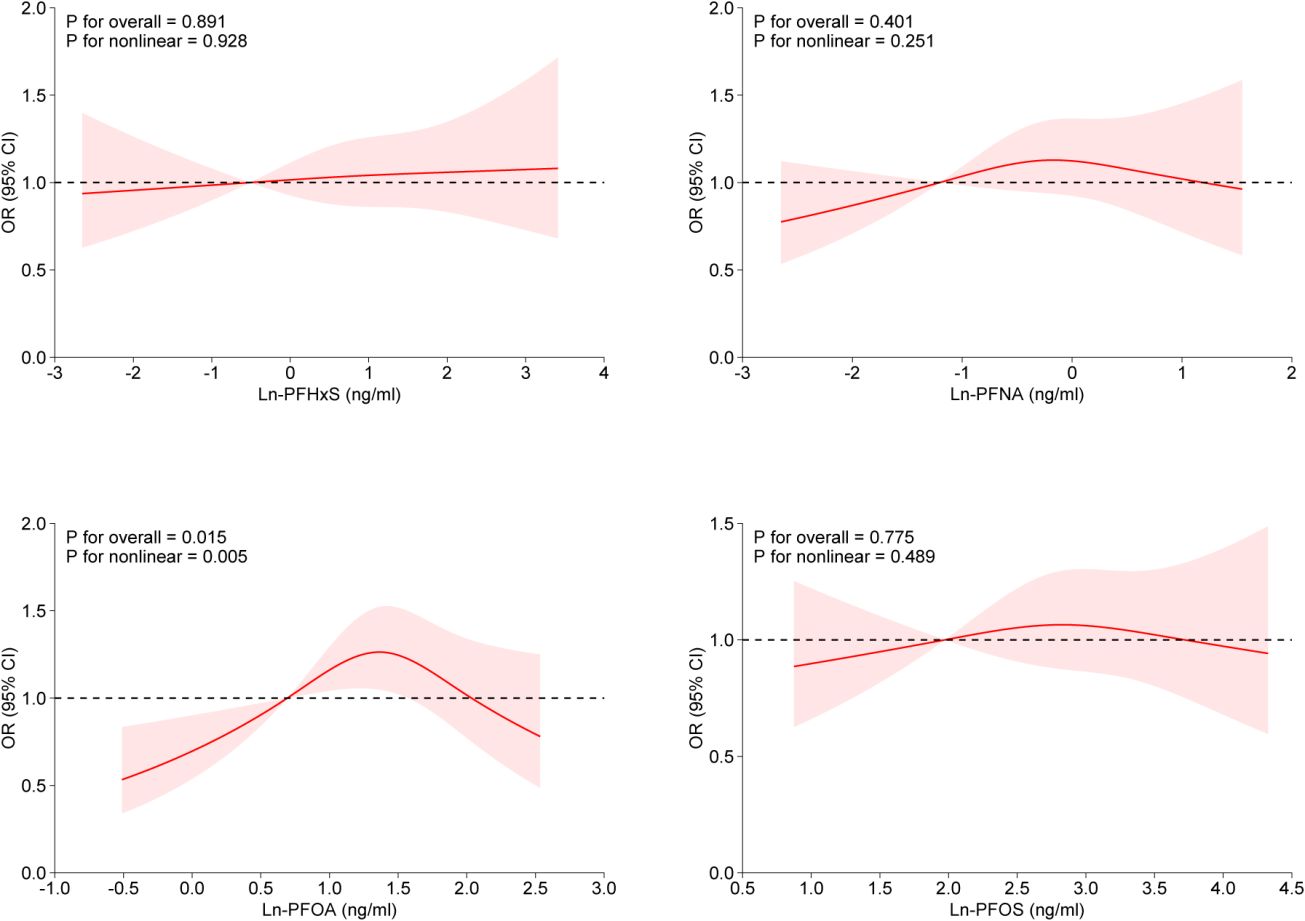
RCS analysis of the relationship between log-transformed PFAS levels and the risk of myopia.

Figure 2 presents the findings related to the dose-response relationship between PFAS exposure and myopia. The results revealed a nonlinear, inverted U-shaped relationship between exposure to PFOA and myopia risk. After adjusting for all relevant covariates, the inflection point on the RCS curve for ln-PFOA was determined to be 0.75 ng/ml. Elevated PFOA exposure levels were linked to a heightened risk of myopia; however, beyond a certain threshold, additional increases in PFOA levels were associated with a reduced likelihood of myopia development.

### 3.5 Mediation analysis

**Fig. 3:**
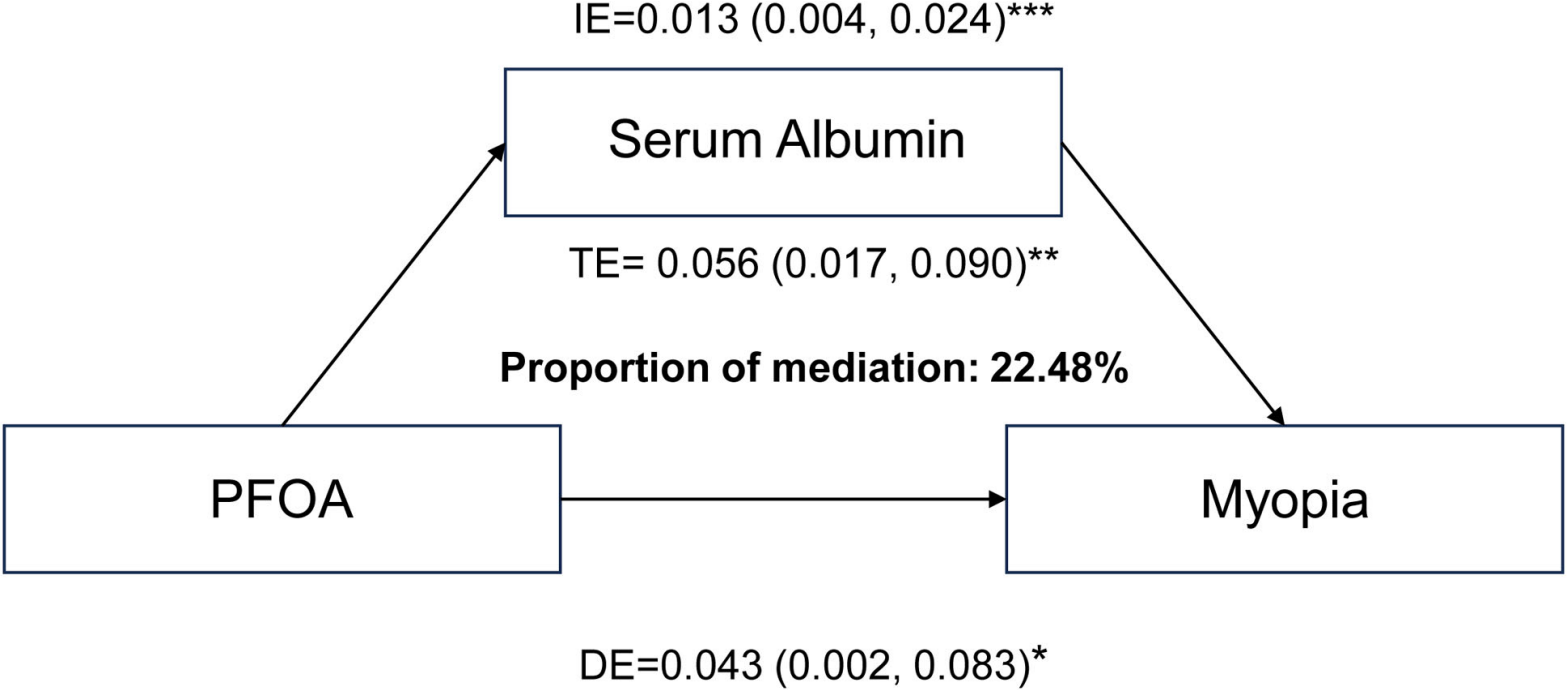
Mediation analysis illustrating effect of serum albumin on interaction between PFOA exposure and myopia risk.

Compared with normal-vision individuals, adolescents with myopia presented with increased serum ALB concentrations (Table 1, P = 0.018). High levels of PFHxS (β: 0.03; 95% CI: 0.02–0.05; P < 0.001), PFOA (β: 0.11; 95% CI: 0.07–0.16; P < 0.001), PFOS (β: 0.11; 95% CI: 0.07–0.14; P < 0.001) were associated with an elevated serum ALB concentration (Table 5). Moreover, the serum ALB level was associated with the risk of myopia (OR: 2.21; 95% CI: 1.42–3.44; P < 0.001). With respect to the first quartile, the fourth quartile of participants presented with a greater myopia risk (OR_Q4_: 1.85; 95% CI: 1.22 to 2.81; P = 0.004) (Table 6). To examine the role of the serum ALB concentration in the relationship between the level of PFOA and myopia risk, we performed a mediation analysis. The results revealed that the serum ALB concentration significantly mediated the relationship between PFOA level and myopia risk, with a positive effect of 22.48% (P = 0.008) (Table 7, Fig. 3).

**Table 5:** Linear regression analysis of the association between PFAS exposure and serum albumin.

	**Crude (β, 95%CI)**	**P-value**	**Model 1 (β, 95%CI)**	**P-value**
PFHxS	0.04 (0.02, 0.06)	**<0.001**	0.03 (0.02, 0.05)	**<0.001**
PFNA	0.01 (−0.02, 0.04)	0.406	−0.01 (−0.04, 0.02)	0.486
PFOA	0.16 (0.12, 0.20)	**<0.001**	0.11 (0.07, 0.16)	**<0.001**
PFOS	0.13 (0.10, 0.17)	**<0.001**	0.11 (0.07, 0.14)	**<0.001**

Note: PFAS, perfluoroalkyl substances; PFHxS, perfluorohexane sulfonate; PFNA, per fluorononanoic acid; PFOA, perfluorooctanoic acid; PFOS, perfluorooctane sulfonic acid

Crude model was adjusted for None.

Model 1 was adjusted for age, sex, race, education level, family income-poverty ratio, BMI, and smoking status of family members.

**Table 6:** Associations between serum ALB concentration and the risk of myopia.

	**OR (95% CI)**	**P value**
Albumin (g/dl)		
Continuous	2.21 (1.42, 3.44)	<0.001
Q1 (<4.2 g/dl)	Reference	
Q2 (4.2–4.4 g/dl)	1.25 (0.71, 2.22)	0.434
Q3 (4.4–4.6 g/dl)	1.45 (0.99, 2.13)	0.054
Q4 (≥4.6 g/dl)	1.85 (1.22, 2.81)	0.004

The model was adjusted for age, sex, race, education level, the family income–poverty ratio, BMI, and the smoking status of family members.

**Table 7:** Mediating effects of serum albumin on association between log-transformed PFAS level and risk of myopia.

**PFAS**	**Direct effects**	**Indirect effects**	**Total effects**	**Mediation ** **proportion (%)**	**P value**

	**β (95% CI)**	**β (95% CI)**	**β (95% CI)**		
PFHxS	−0.005 (−0.032, 0.023)	0.005 (0.001, 0.010) **	0.000 (−0.027, 0,027)	−15.62%	0.964
PFNA	0.027 (−0.009, 0.067)	−0.001 (−0.005, 0.003)	0.026 (−0.011, 0.066)	−2.87%	0.660
PFOA	0.043 (0.002, 0.083) *	0.013 (0.004, 0.024) **	0.056 (0.017, 0.090) **	22.48%	**0.008**
PFOS	−0.001 (−0.051, 0.036)	0.014 (0.004, 0.026) ***	0.012 (−0.029, 0.045)	43.77%	0.500

Abbreviations: PFHxS, perfluorohexane sulfonate; PFNA, perfluorononanoic acid; PFOA, perfluorooctanoic acid; PFOS, perfluorooctane sulfonic acid.

In each model, adjustments were made for factors such as age, sex, ethnicity, educational attainment, the family’s income–poverty ratio, BMI, and the smoking habits of family members. *P<0.05; **P<0.01; ***P<0.001; **P<0.01; *P<0.05

### 3.6 Sensitivity analysis

Considering that studies have shown that CL use in young populations may have a significant effect on PFAS-related body burns [44], a sensitivity analysis was performed. The sensitivity analysis revealed comparable results with our main results (Table S2). After excluding participants who did not wear CLs, compared with the first quartile, the second (Q2) and fourth (Q4) quartiles of PFOA were significantly associated with a greater risk of myopia (OR_Q2_: 1.71; 95% CI: 1.09–2.68; P = 0.020, OR_Q4_: 1.74; 95% CI: 1.17–2.57; P = 0.007). No associations were found between PFHxS, PFNA, or PFOS levels and myopia risk.

## 4. Discussion

The results of this study showed that PFOA exposure is related to the risk of myopia. Furthermore, these results could be explained in part by the effect of serum ALB. In addition, a nonlinear, inverted U-shaped relationship was identified between PFOA exposure and myopia risk in adolescents from the NHANES survey between 1999 and 2008. The results of this study highlight concerns about the possible link between myopia and PFOA exposure.

While research on the effects of PFASs on eye health is limited, it is essential to be vigilant about potential harm to the eye. Lee et al. [54] used zebrafish larvae as an experimental model and discovered that exposure to PFASs, particularly PFOS, led to impairments in visual function, especially phototactic and optomotor responses. Kang et al. [55] revealed that individuals who wore CLs had a significantly greater overall burden of PFAS in their systems than nonusers did. Moreover, the FDA has approved new PFAS-containing eye drops as a treatment for dry eye disease [56]. Exposure to PFASs may also occur in individuals undergoing eye surgeries, such as retinal detachment operations, due to the application of liquid perfluorocarbon in these procedures [57]. However, although few studies have investigated the potential exposure of PFASs to the eyes, further research in this field is needed.

Exposure to PFAS, especially for PFOA may enhance the risk of myopia development via oxidative stress-mediated inflammation and consequent scleral remodeling [41, 42]. Specifically, PFAS exposure triggers the production of reactive oxygen species (ROS), which can damage cellular components, including DNA, proteins, and lipids [43]. This damage may contribute to the pathogenesis of myopia by affecting ocular tissues such as the sclera and retina [20]. Elevated levels of malondialdehyde (MDA), a marker of lipid peroxidation, have been observed in both PFOA-exposed animal models and myopic patients [44]. Chronic inflammation, characterized by elevated cytokines like IL-6 and TNF-α, has been implicated in scleral remodeling and thinning, processes central to myopia development [45]. Studies have shown that inflammatory mediators such as IL-6 promote matrix metalloproteinase (MMP) activity, further supporting this mechanism [46, 47]. Additionally, PFAS compounds are known endocrine disruptors, interfering with hormonal pathways that regulate growth and development. During critical periods of eye development, disruptions in these pathways could potentially influence refractive error outcomes, including myopia. Collectively, these findings suggest a potential link between PFAS exposure and myopia through oxidative stress and inflammation pathways.

Our research revealed a nonlinear, inverted U-shaped relationship between the progression of myopia and exposure to PFOA. PFOA has been shown to contribute to neurobehavioral deficits, as tit can lead to alterations in the expression of neurotransmitters [58]. Within the retina, dopamine acts as a vital neurotransmitter, regulating key processes, including retinal development, visual signal transmission, and the modulation of refractive errors [59]. Although how PFASs affect myopia development is still unknown, experimental studies have indicated that PFASs can accumulate in the brain and may disturb dopamine [60, 61]. Prior studies have shown that PFAS can disrupt the dopamine and steroid hormone balance in Bank voles [62], which may indicate a potential link between PFAS and myopia. PFASs can penetrate the brain either by compromising tight junctions—allowing penetration into brain tissue—or by adhering to transport proteins to breach the plasma membrane [63, 64]. This penetration can result in neurotoxic effects that alter dopamine signaling [65]. Foguth et al. demonstrated that developmental exposure to PFASs leads to selective depletion of total brain dopamine in amphibian sentinel species [66]. Owing to the absence of dopamine data in the current study, we were unable to definitively ascertain its mediating influence on the relationship between myopia and PFAS. The precise mechanism through which PFAS influences the development of myopia remains to be further investigated.

Subgroup analyses revealed significant differences in the PFAS-related myopia risk across different demographics. Given that adolescents may be more vulnerable to both exogenous and endogenous hormone fluctuations than adults are, we performed a sex-based analysis [67]. The results revealed a strong correlation between PFOA exposure and the risk of myopia in female participants, potentially due to the demographics of the study subjects, as women tend to have higher estradiol levels.

Reports have suggested that PFASs disrupt enzyme synthesis and steroidogenesis, demonstrating a strong binding affinity for estrogen receptors, leading to higher concentrations of PFOA in the blood of women than in that of men [68, 69]. This increased PFOA exposure is positively correlated with chronic inflammation and oxidative stress, which may serve as a potential underlying mechanism by which women are more prone to developing myopia or near-vision issues.

The results of the mediation analysis suggested that the serum ALB concentration may positively influence the association between PFOA exposure and myopia risk, indicating that elevated serum ALB levels could be a potential risk factor for myopia. The precise underlying mechanism of this mediation remains unclear, however. A plausible explanation is that serum ALB, as the predominant protein in plasma, has strong binding affinity for PFASs and thus serves as their primary transporter in the bloodstream [70, 71]. Serum ALB serves as a critical marker of systemic metabolic health, and recent research has highlighted the close relationship between metabolic abnormalities and myopia [72]. Additionally, serum ALB plays a key role in transporting various nutrients throughout the body. Higher socioeconomic status is often correlated with improved medical care and elevated serum ALB levels; however, it may also increase the risk of myopia due to prolonged near-work activities. Individual genetic susceptibility could render certain individuals more prone to developing myopia at specific serum ALB concentrations. It is important to note that no definitive evidence exists to establish causality between serum ALB levels, PFOA, and myopia. Their association may instead be mediated by intricate biological mechanisms and influenced by confounding factors. Further, robust studies are needed to explore the mediating role of serum ALB in the relationship between PFAS exposure and eye health outcomes.

In this study, we, for the first time, investigated the correlation between serum PFAS levels and myopia in adolescents. To mitigate bias from oversampling, we employed weighted estimates and adjusted for various confounders, thus increasing the reliability and precision of our statistical results. Furthermore, we performed a sensitivity analysis to confirm the reliability of our results, demonstrating consistency across various subgroup analyses and different modeling strategies. After the impact of PFAS caused by CL wearing was excluded, this robustness was maintained in the results.

This study also has certain limitations that should be considered when interpreting the findings. First, the cross-sectional nature of our study prevented an analysis of the long-term impact of PFASs on myopia or the establishment of causation. Larger-scale epidemiological investigations are needed to further evaluate the correlations between serum PFASs, notably PFOA, and the risk of myopia among children. Second, despite adjusting for multiple confounding factors, we could not eliminate the influence of certain other confounders, such as the time spent outdoors, the use of atropine eye drops, or orthokeratology, which may have affected the results.

## 5. Conclusion

The results of our study revealed that serum concentrations of PFOA, particularly in adolescents, are significantly associated with the risk of myopia. Furthermore, the serum ALB concentration may play a mediating role in this relationship. These findings contribute to the understanding of the environmental factors influencing myopia and highlight the potential toxicological impact of PFASs on eye health. Further research is needed to confirm these findings and explore them in greater depth.

## Supplementary information

**Additional file 1:** **Table S1.** Limits of detection for PFAS substances concentrations across cycles.

**Additional file 2:** **Table S2.** Sensitivity analysis (n = 1829).

## Declarations

### Availability of data and materials

The data will be made available upon request.

The code associated with this study can be obtained from the following URL: (https://github.com/Cabri-pku/PFAS_MYOPIA/tree/main)
