# Supplementary material for: Associations between per- and polyfluoroalkyl substance exposure and the prevalence of myopia in adolescents: the mediating role of serum albumin
Source: Environ Health Prev Med. 2025 Jun 27;30:50. doi: 10.1265/ehpm.25-00023 (PMC12206666; doi:10.1265/ehpm.25-00023)
Supplement: Supplementary file 1 — Additional file 1: Table S1. Limits of detection for PFAS substances concentrations across cycles. [file ehpm-30-050-s001.docx]

**Table S1. Limits of detection for PFAS substances concentrations across cycles.**

| Cycle | PFOA | PFOS | PFNA | PFHxS |
| --- | --- | --- | --- | --- |
| 1999-2000 | 0.07 | 0.14 | 0.07 | 0.07 |
| 2003-2004 | 0.1 | 0.4 | 0.1 | 0.3 |
| 2005-2006 | 0.1 | 0.2 | 0.1 | 0.1 |
| 2007-2008 | 0.1 | 0.2 | 0.082 | 0.1 |

Note: PFAS: perfluoroalkyl substances; PFHxS, perfluorohexane sulfonate; PFNA, per fluorononanoic acid; PFOA, perfluorooctanoic acid; PFOS, perfluorooctane sulfonic acid
