# Supplementary material for: Associations between per- and polyfluoroalkyl substance exposure and the prevalence of myopia in adolescents: the mediating role of serum albumin
Source: Environ Health Prev Med. 2025 Jun 27;30:50. doi: 10.1265/ehpm.25-00023 (PMC12206666; doi:10.1265/ehpm.25-00023)
Supplement: Supplementary file 2 — Additional file 2: Table S2. Sensitivity analysis (n = 1829). [file ehpm-30-050-s002.docx]

|  | Crude (OR, 95%CI) | P-value |
| --- | --- | --- |
| PFHxS |  |  |
| ln-PFHxS | 0.98 (0.85, 1.12) | 0.719 |
| Q1 (<1.2) |  |  |
| Q2 (1.2-2.3) | 1.12 (0.74, 1.71) | 0.573 |
| Q3 (2.4-4.6) | 0.86 (0.52, 1.40) | 0.533 |
| Q4 (≥4.6) | 1.12 (0.74, 1.70) | 0.569 |
| PFNA |  |  |
| ln-PFNA | 1.13 (0.92, 1.40) | 0.246 |
| Q1 (<0.6) |  |  |
| Q2 (0.6-0.902) | 1.30 (0.81, 2.09) | 0.268 |
| Q3 (0.902-1.312) | 1.01 (0.62, 1.67) | 0.969 |
| Q4 (≥1.312) | 1.34 (0.80, 2.21) | 0.256 |
| PFOA |  |  |
| ln-PFOA | 1.30 (0.99, 1.70) | 0.059 |
| Q1 (<3.0) |  |  |
| Q2 (3.0-4.0) | **1.71 (1.09, 2.68)** | **0.020** |
| Q3 (4.0-5.4) | 1.43 (0.91, 1.25) | 0.118 |
| Q4 (≥5.4) | **1.74 (1.17, 2.57)** | **0.007** |
| PFOS |  |  |
| ln-PFOS | 1.14 (0.88, 1.47) | 0.320 |
| Q1 (<10.4) |  |  |
| Q2 (10.4-15.7) | 1.00 (0.53, 1.89) | 0.989 |
| Q3 (16.0-24.1) | **1.63 (1.01, 2.66)** | **0.049** |
| Q4 (≥24.1) | 1.23 (0.73, 2.09) | 0.428 |

**Table S2.** Sensitivity analysis (n = 1829).

Note: PFAS: perfluoroalkyl substances; PFHxS, perfluorohexane sulfonate; PFNA, per fluorononanoic acid; PFOA, perfluorooctanoic acid; PFOS, perfluorooctane sulfonic acid

Model was adjusted for age, sex, race, education level, family income-poverty ratio, BMI, and smoking status of family members.
